# Patient Participation in Nursing Handover in a Coronary Intensive Care Unit: A Qualitative Study

**DOI:** 10.1002/nop2.70802

**Published:** 2026-09-08

**Authors:** Grazielle Rezende da Silva dos Santos, Sabrina da Costa Machado Duarte, Juliana Faria Campos, Rafael Celestino da Silva

**Affiliations:** ^1^ Federal University of Rio de Janeiro, Anna Nery School of Nursing Rio de Janeiro Rio de Janeiro Brazil

**Keywords:** intensive care units, nursing, patient handoff, patient participation, patient safety

## Abstract

**Aim:**

To explore the perceptions of patients and nursing professionals regarding patient participation in nursing handover in a coronary intensive care unit.

**Design:**

An exploratory qualitative study grounded in patient‐centered care and informed by the continuum of patient engagement.

**Methods:**

The study was conducted in a coronary intensive care unit of a hospital in Rio de Janeiro, Brazil. Participants were 22 nursing professionals and 16 hospitalized patients. Data were collected between October 2023 and May 2024 through individual semi‐structured interviews and analysed using inductive thematic content analysis. The study was reported according to the COREQ checklist.

**Results:**

Nursing professionals recognized that patient participation in handover could improve communication and patient safety by allowing patients to clarify, correct, or complement information about their care. However, they also expressed concerns regarding confidentiality, privacy, emotional burden, technical language, increased handover duration, and patients' clinical or cognitive conditions. Although patients valued receiving information and opportunities to communicate with professionals, many did not perceive themselves as legitimate participants in the handover process and preferred a passive role unless explicitly invited to contribute.

**Conclusions:**

Bedside handover alone does not ensure meaningful patient participation. Transitioning from bedside to participatory handover requires organizational readiness, effective communication strategies, and flexible approaches that respect patients' preferences, clinical condition, health literacy, and willingness to participate. Rather than a universal intervention, participatory handover should be implemented as an individualized opportunity to strengthen communication and patient‐centred care.

**Implications for the Profession and/or Patient Care:**

Healthcare organizations seeking to implement participatory nursing handover should combine staff training, structured communication processes, plain‐language communication, explicit invitations to patients, and privacy safeguards. These strategies may facilitate the transition from professionally centred bedside communication to collaborative, patient‐centred handover while maintaining patient safety.

**Impact:**

This study distinguishes bedside handover from participatory handover by demonstrating that patients' presence during bedside communication does not necessarily translate into meaningful engagement. The findings highlight organizational, cultural, and communication factors that should guide the implementation of participatory handover in intensive care settings.

**Reporting Method:**

The study report was guided by the COREQ checklist.

**Patient or Public Contribution:**

No Patient or Public Contribution. Patients participated as research participants but were not involved in study design, conduct, analysis, interpretation, or manuscript preparation.

## 1 Introduction

Patient safety is a global priority in healthcare, particularly because preventable adverse events remain frequent and may result in serious harm (World Health Organization 2021). According to the World Health Organization (2021), in high‐income countries around 10% of hospitalized patients are exposed to adverse events, while in low‐ and middle‐income countries, this proportion may reach 25%, with an estimated 134 million incidents per year and approximately 2.6 million deaths attributable to unsafe care.

In response to this scenario, the World Health Organization proposed the Global Patient Safety Action Plan 2021–2030, which identifies patient and family engagement as a key strategy for improving the safety of healthcare systems and reducing avoidable harm (World Health Organization 2021).

In this study, patient engagement is understood as an active partnership among patients, healthcare professionals, and families across different levels of care. This perspective is grounded in the framework proposed by Carman et al. (2013), in which engagement is conceptualized as a continuum, ranging from consultation and information sharing to active partnership and shared decision‐making (Carman et al. 2013). At the level of direct care, this continuum includes patients receiving understandable information, asking questions, expressing concerns, contributing experiential knowledge and, when appropriate, participating in decisions about their care (Carman et al. 2013). However, engagement is influenced by patient‐related, organizational, social and political factors, including health literacy, beliefs, clinical condition, professional culture, institutional policies and broader norms regarding patients' roles in healthcare (Carman et al. 2013).

In Brazil, patient engagement has only recently become an explicit priority within national patient safety policies (Cruz and Pedreira 2020). Brazilian healthcare institutions continue to face challenges in implementing patient‐centred care, including the persistence of biomedical traditions, hierarchical communication practices, and limited patient involvement in care decisions (World Health Organization 2021; Lima and Machado 2021). These contextual elements can influence how professionals and patients understand participation, particularly in hospital environments where clinical complexity and organizational routines may reinforce professional‐centred communication.

Nursing handover during shift changes is a critical moment for communication and continuity of care. It involves the transfer of information, responsibility, and accountability among nursing professionals and is particularly relevant in intensive care settings, where communication failures may directly compromise patient safety (Kim and Seomun 2020; AlAmrani 2022). Bedside handover has been recommended as a strategy to improve information accuracy, allow direct patient visualization, and support safer care transitions (Anshasi and Almayasi 2024; Bressan et al. 2019; Hada and Coyer 2021; Tobiano et al. 2018). When patients are invited to participate, they may help clarify symptoms, correct inaccurate information, report concerns, ask questions, and better understand their care plan (Anshasi and Almayasi 2024; Bressan et al. 2019; Hada and Coyer 2021; Tobiano et al. 2018; Street et al. 2022).

Nevertheless, patient participation in nursing handover is neither automatic nor universally appropriate. Evidence has shown that patients' willingness and ability to participate vary according to their preferences, health condition, emotional readiness and understanding of the handover process (Anshasi and Almayasi 2024; Bressan et al. 2019; Street et al. 2022; Lantz et al. 2023). Nursing professionals may also express concerns about confidentiality, privacy, technical language, increased duration of handover, emotional burden for patients and uncertainty about which information should be shared at the bedside (Tobiano et al. 2018; Jimmerson et al. 2021; Figueiredo et al. 2019; Chien et al. 2022). Consequently, simply performing handover at the bedside does not necessarily result in meaningful patient participation. Instead, participation should be understood as a context‐sensitive practice that requires preparation, ethical judgement and adaptation to patients' needs and clinical conditions.

Although international literature has examined bedside handover and patient participation, most studies have been conducted in high‐income countries, especially in North America, Europe, and Australia (Street et al. 2022; Lantz et al. 2023; Jimmerson et al. 2021). Consequently, little is known about how these practices are perceived in Latin American intensive care settings, where sociocultural, organizational, and healthcare‐system characteristics differ substantially from those of countries in which most evidence has been generated. Understanding these perceptions is necessary to support contextually appropriate, ethically sensitive, and clinically feasible strategies for involving patients in nursing handover.

Although bedside handover has frequently been associated with greater patient involvement, it remains unclear whether patients' presence during handover necessarily translates into meaningful participation (Ghosh et al. 2025). This distinction has received limited attention, particularly in intensive care settings, where ethical, organizational, and communication challenges may shape both professionals' practices and patients' willingness to engage. Exploring these perceptions may help clarify the conditions under which bedside handover can support genuinely participatory communication.

## 2 Objective

This study aims to explore the perceptions of patients and nursing professionals regarding patient participation in nursing handover in a coronary intensive care unit.

## 3 Methods

### 3.1 Study Design and Theoretical Framework

This was an exploratory qualitative study conducted through individual semi‐structured interviews with nursing professionals and hospitalized patients. An exploratory qualitative design was considered appropriate because the study sought to understand how participants perceived and interpreted patient participation in nursing handover within their everyday clinical context. The report was guided by the Consolidated Criteria for Reporting Qualitative Research (COREQ).

The results of these interviews are part of a doctoral thesis in nursing completed in 2025 by the first author of this article, the general objective of which was to develop a nursing handover model with patient participation. A preliminary analysis of the interview results was presented in abstract form at scientific events focused on research and patient safety held in 2025, while the final analysis of the dataset—interpreted through the adopted theoretical framework—is being published for the first time in this scientific article.

The study was grounded in the framework of patient‐centred care, which provided the conceptual foundation for understanding patient participation as a partnership between professionals and patients rather than solely as information sharing (Carman et al. 2013). For the interpretation of the findings, we also used Carman et al.'s framework of patient and family engagement, particularly the continuum of engagement at the level of direct care. This framework supported the analysis of how patients and nursing professionals perceived different forms and levels of participation in handover, ranging from receiving information and listening passively to asking questions, complementing information, and sharing responsibility in care‐related communication (Carman et al. 2013).

### 3.2 Setting

The study was conducted in a coronary intensive care unit (ICU) located in a hospital in Rio de Janeiro, Brazil. The unit has eight beds for intensive cardiology care to patients with acute coronary syndromes and other highly complex clinical conditions, as well as one bed for patients in the preoperative and postoperative period of cardiac surgery. The average length of stay of adult patients in the unit is approximately 6 days.

Nursing handover takes place twice a day, at 7:00 a.m. and 7:00 p.m., during shift changes. In the study setting, handover is usually performed at the bedside and supported by a structured communication instrument used by nursing professionals. However, despite occurring at the bedside, the process is predominantly professional‐centred and does not systematically include active patient participation. The setting was selected because communication failures during nursing handover may have direct implications for patient safety, while many coronary ICU patients remain conscious and clinically stable enough to report their experiences of communication.

### 3.3 Sampling, Inclusion and Exclusion Criteria, and Recruitment

Participants were nursing professionals working in the coronary ICU and patients hospitalized in the same unit. A purposive sampling strategy was used to ensure the inclusion of participants with direct experience of the phenomenon under investigation.

The nursing staff of the unit consisted of professionals distributed across work shifts. A typical shift included a head nurse, two staff nurses, and five nursing technicians, working 12‐h shifts followed by 60 h off. However, the total eligible nursing staff during the study period consisted of 53 professionals. Of these, 22 participated in the study: eight nurses and 14 nursing technicians. Nurses were responsible for team leadership, care management and comprehensive care for critically ill patients, whereas nursing technicians provided direct care activities of lower complexity under nurses' supervision.

Nursing professionals were eligible if they were members of the nursing team, were working in the unit during the data collection period and participated in nursing handover. Professionals temporarily reassigned from other hospital units were excluded. Of the 53 professionals eligible at the start of the study, ten declined to participate and 21 were not approached—either because their employment with the institution ended during the study period or because they had been reassigned from other hospital units.

Patients were eligible if they were aged 18 years or older, hospitalized in the coronary ICU for at least 48 h and had experienced at least four nursing handovers. Patients were excluded if they were unable to speak, had cognitive impairment, or presented clinical instability at the time of data collection. These criteria were adopted to ensure that participants were able to understand the invitation, provide informed consent and describe their perceptions without compromising their clinical safety or well‐being. Recruitment occurred through direct individual invitation by the researcher in the clinical setting. Before recruitment, the researcher completed a familiarization period in the unit to understand the work dynamics, present the study to the team, identify eligible participants and clarify doubts. Patients were invited only after confirmation of clinical stability and availability. No flyers or e‐mail invitations were used. Participation was voluntary, and refusal did not affect professional activities or patient care. At the end of the recruitment phase, 16 patients were included in the study, 16 were excluded due to their clinical conditions, and one declined to participate in the research.

Data collection ended when empirical saturation was reached, that is, when additional interviews no longer generated substantially new concepts or insights relevant to the study objective. During preliminary analysis, the research team observed that the interviews provided sufficient depth and recurrence of meanings to understand the phenomenon under investigation, with no substantially new elements emerging from additional interviews. This analysis of the point of saturation was conducted separately for the interview corpus of nursing professionals and patients.

### 3.4 Data Collection

Data were collected through semi‐structured individual interviews conducted between October 2023 and May 2024 by G.R.S.S., female, nurse, Master of Science in Nursing and doctoral researcher at the time of the research. She has experience in nursing handover in intensive care and qualitative interviewing. The researcher had no previous relationship with the participants.

Two semi‐structured interview guides were developed, one for nursing professionals and another for patients, based on the study objectives and the theoretical framework. The guide for nursing professionals explored perceptions of the current handover model, patient participation in handover, communication structure during shift changes, implications for patient safety, perceived benefits, barriers, ethical concerns, and possible strategies for implementation. Sociodemographic and professional variables were also collected, including age, sex, professional category, time since graduation, time working in the unit, and other employment relationship.

The patient interview guide explored patients' perceptions of communication among nursing professionals, their understanding of nursing handover, the type of information they considered necessary to understand their health condition, their willingness or unwillingness to participate in handover, and perceived facilitators and barriers to participation. Patient characteristics collected included age, sex, education, length of hospitalization, and clinical diagnosis or condition. The complete interview guide used with the professionals and patients who participated in the study is presented in the Supporting Information of this article.

The interview guides were developed specifically for this study and were informed by the study objective, patient‐centred care principles and the patient engagement framework proposed by Carman et al. (2013). The interview guides were pilot tested with nursing professionals and patients with similar profiles in a clinical ward of the same hospital. The pilot test aimed to assess the clarity, relevance and sequence of the questions. Based on the pilot, minor changes were made to improve comprehensibility and conversational flow rather than to modify the content of the interview domains.

Professional interviews were conducted in a private room within the unit, at times agreed with participants and without disrupting care activities. Patient interviews were conducted at the bedside during periods of clinical stability and absence of procedures and direct care activities, and according to patients' availability and willingness.

All interviews were conducted in Portuguese, audio‐recorded with participants' consent, and transcribed verbatim in Portuguese. Interviews with nursing professionals lasted between 9 and 20 min, with a mean duration of 15 min. Interviews with patients lasted between 7 and 15 min, with a mean duration of 10 min.

### 3.5 Data Analysis and Rigour

Data were analysed using inductive thematic content analysis (Vaismoradi et al. 2013). This method was selected because it allows patterns of meaning to emerge inductively from participants' accounts while maintaining analytical transparency and methodological rigour. After deriving the themes through this inductive model, the patient engagement framework was applied as an interpretive lens for the findings.

All transcripts were analysed in Portuguese, the original language of the interviews, to preserve participants' meanings, cultural nuances and contextual expressions. Translation into English occurred only after the analytical structure had been defined. Selected quotations were translated from Portuguese into English by the authors and checked for semantic and cultural equivalence by a bilingual researcher. The translated excerpts presented in the Findings section are verbatim translations, not summaries.

The analytical process followed three main stages (Vaismoradi et al. 2013). First, in the pre‐analysis stage, the transcripts were read repeatedly to allow immersion in the data and identification of initial impressions related to the research objective. Second, in the exploration stage, meaningful excerpts were identified and coded as thematic recording units. These recording units were then grouped according to semantic similarity and relevance to the phenomenon, resulting in 15 meaning units. Third, in the treatment and interpretation stage, the meaning units were compared, merged, or separated according to conceptual proximity, recurrence, explanatory strength, and coherence with the theoretical framework. This process resulted in four final themes.

Throughout the analytical process, movement between the data, codes, meaning units and emerging themes was iterative rather than strictly linear.

The complete corpus comprised 450 thematic recording units distributed across 15 meaning units. The frequency of recording units was used only to support transparency in the analytical process and to describe the structure of the corpus; it was not interpreted as a measure of statistical representativeness. Less frequent units were not automatically excluded. They were retained whenever they provided conceptually important insights, irrespective of their frequency.

Data organization and coding were performed manually using Microsoft Word and Excel. Audio recordings were transcribed manually and checked against the original audio files for accuracy before analysis.

To enhance rigour, the analysis was conducted collaboratively and cross‐validated by the research group. The initial coding and grouping of recording units were performed by the G.R.S.S. doctoral researcher and then reviewed by senior researchers R.C.S. and J.F.C., who has experience in qualitative research, patient safety and nursing handover. The research group compared codes, reviewed the allocation of recording units into meaning units, discussed convergences and divergences, and refined the thematic structure until consensus was reached. Reflexive discussions among the research team were also used to challenge preliminary interpretations and reduce the influence of individual assumptions during theme development. This process strengthened credibility, analytical consistency and interpretive depth.

Although interviews with nursing professionals and patients were initially analysed separately because they represented different positions in the handover process, the final interpretation considered convergences and tensions between both groups. In the Findings section, themes are identified according to whether they were derived mainly from nursing professionals, patients, or both.

### 3.6 Ethical Considerations

The study was approved by the Research Ethics Committee of the participating institution, under approval number 5.203.363 and Certificate of Presentation for Ethical Review 52279221.0.3001.5257. All participants signed an informed consent form before data collection.

Additional precautions were adopted because patients were hospitalized in a coronary intensive care unit. Only patients who were clinically stable, able to communicate, and without cognitive impairment were invited. Interviews were scheduled according to patients' availability and were not conducted during procedures, direct care activities, clinical deterioration, pain, fatigue, or emotional discomfort. Patients were informed that participation was voluntary and that they could decline, pause, or withdraw at any time without any consequence for their care.

Confidentiality was ensured through alphanumeric codes. Nurses were identified as “NUR”, nursing technicians as “TEC”, and patients as “PAT”, followed by the sequential interview number. Identifying information was removed from transcripts and quotations.

## 4 Findings

### 4.1 Participant Characteristics

Thirty‐eight participants were included in the study: 22 nursing professionals and 16 hospitalized patients. The nursing professional group consisted of eight nurses and 14 nursing technicians. Most professionals were women (64%), predominantly aged between 31 and 50 years (64%), with a mean age of 47 years (SD ± 9.51), ranging from a minimum of 31 to a maximum of 61 years. Furthermore, they had more than 10 years since graduation (86%) and had an average professional experience of 22 years, had worked in the coronary intensive care unit for < 5 years (55%), and did not report another employment relationship (55%). Among the 16 patients, most were women (63%) and were aged over 50 years (87%), with a mean age of 58 years (SD ± 13.44) ranging from a minimum of 27 to a maximum of 78 years. The group was characterized by low formal education, with a predominance of incomplete primary education (63%), which may have influenced how participants understood nursing handover and their perceived legitimacy to participate in this process.

### 4.2 Thematic Structure

The analysis generated 450 thematic recording units, which were grouped into 15 meaning units and then organized into four themes. The themes represent the final interpretative structure derived from the analytical process. The first three themes were mainly derived from interviews with nursing professionals, whereas the fourth theme was derived from interviews with patients. Each theme comprised conceptually related meaning units that emerged through iterative comparison and abstraction during the analytical process (Vaismoradi et al. 2013).

#### 4.2.1 Theme 1. Nursing Professionals' Perceptions of Current Bedside Handover

This theme was derived mainly from nursing professionals' accounts and represented 94 thematic recording units. In the study setting, nursing handover was already conducted at the bedside and supported by a structured communication instrument. Professionals considered this model to enhance communication accuracy because it allowed them to visualize the patient, check devices, and complement information during the shift transition.

One nurse described bedside handover as a strategy that helped prevent safety incidents:

This handover model helped reduce these incidents because you can see the patient. The patient is exposed to risks, such as falling from the bed, as we observed today. (NUR 04)

The structured handover script was also described as a memory aid that supported completeness of communication:

I think handover is more effective now. We have a structured handover script that ensures all important points about the patient are covered, and it is conducted at the bedside. (NUR 05)

There is a form we fill out, and sometimes when we are talking, something might be overlooked and this form has strategic points that help memory. (NUR 06)

Although handover occurred at the bedside, patients were not systematically included as active participants. Even so, professionals recognized that patients could complement, correct or clarify information, especially when information was missing, inaccurate or incomplete.

Most of the time, the patient knows everything that is happening to them. They know their diseases and their treatment. It has happened to me that a patient corrected me or added information. I think that is valid. (NUR 07)

A nursing technician similarly emphasized that patients could contribute to preventing adverse events by reporting information that might not have been captured during admission or documentation:

Sometimes we discover certain things by talking directly with the patient, for example, allergies. By involving the patient and asking them, we could obtain this information and prevent an adverse event or greater harm. (TEC 01)

From their professionals' perspective, when patients understand what is happening and are included in communication, they may feel better informed and more secure.

When the patient feels involved, I think it becomes much calmer, because when you do not have information, you become more anxious. Anxiety leads to many other problems, not only emotional ones, but also clinical problems, such as increased blood pressure, stress and headache. When you are more informed, your anxiety level decreases. (NUR 05)

#### 4.2.2 Theme 2. Barriers to Implementing Patient Participation in Handover

This theme was also derived mainly from nursing professionals' accounts and represented 135 thematic recording units. Professionals expressed ambivalence regarding the implementation of patient participation in handover. Some were favourable to the idea, particularly if implementation occurred gradually and within a structured process, whereas others expressed resistance, uncertainty, or concern.

I would accept it… I would have nothing against it. Anything that benefits the patient, we have to promote. It's always important to improve care, to put ourselves in the patient's shoes. (NUR 02)

I am in favor, as long as it is introduced gradually and in a disciplined way. (TEC 05)

However, resistance was also evident:

It's something very new. And anything that is very new causes strangeness and resistance. I think it would be very good, but I don't even know if such a model exists anywhere. (NUR 06)

I don't agree, I don't think it's viable. It brings embarrassment and greater concern when the patient doesn't need to be worried. They need to feel safe. (TEC 10)

Communication emerged as one of the principal barriers to implementation. Professionals recognized that acronyms, biomedical terminology and unexplained clinical expressions could confuse patients, increase anxiety or generate misunderstandings.

When you do bedside handover, you cannot use technical terms because patients don't understand them, unless you explain what it means. (NUR 04)

Once a patient was shocked because he thought he was HIV positive. You have to be very careful. We use acronyms that patients don't understand, which can increase stress. (NUR 05)

When patients participate, we should use terminology they can understand, speak in plain language. There is no point in using technical words that they will not know. (TEC 13)

Professionals also questioned whether all information should be shared at the bedside. Some considered that certain content could cause emotional distress, especially when related to bleeding, severe prognosis, uncertain medical decisions or complex clinical information.

When you say in handover that the patient bled a lot, even if the patient is oriented and you explain that the loss will be replaced, depending on the person, this can make them nervous and anxious. (NUR 04)

It is confusing for a lay patient. We, as health professionals, have worked with this for a long time. The patient has no knowledge about that issue, so some information could make them even more confused. (NUR 06)

Sometimes you end up sharing things that are not inherent to the patient, things they don't need to know, won't fully understand, or won't be interested in. It may just confuse or expose them. (TEC 10)

Professionals also identified organizational and contextual barriers, including increased handover duration, the physical characteristics of the ICU, loss of privacy, exposure of sensitive information and patients' clinical or cognitive conditions. The open structure of the unit and proximity between beds were perceived as obstacles to confidential communication.

Handover has to be dynamic. We need to pass information in a concise and efficient way because care needs to continue. The outgoing staff often need to leave quickly. Extending handover makes things harder for those leaving and those arriving. (NUR 03)

I think it's too much exposure for patients, especially in the ICU, where beds are side by side with only a screen in between them. You end up exposing their disease and history, which has a negative side. (NUR 07)

We often deal with disoriented patients because of confinement syndrome. They stay here for a long time and can become anxious or confused, which could make participation harder. (TEC 01)

#### 4.2.3 Theme 3. Enablers for Implementing Patient Participation in Handover

This theme, derived mainly from nursing professionals' accounts, represented 84 thematic recording units. Professionals emphasized that successful implementation would require a structured model, clear criteria about what information should be shared, staff training, and strategies to support patient involvement.

A recurrent issue was the need to define which information should be communicated at the bedside and which should be discussed separately among professionals. Participants did not agree on whether patients should hear all information, but they converged on the need for clear boundaries and guidance

Sometimes, during handover, we mention something important for my care delivery, but it's not the right moment for the patient to know, because a medical decision still has to be made. Some things shouldn't be told at that moment, but they are important for nursing care. So there are conflicts. (TEC 05)

For the patient's psychological well‐being, hearing repeatedly, “terminal illness” twice a day may be harmful. In those cases, I prefer to discuss the history more discreetly or point it out separately. (NUR 07)

This really needs to be worked on: what can and cannot be said during bedside handover. Not everything is appropriate for the patient to hear. (NUR 05)

Training was identified as a necessary condition for implementation. Professionals reported that they would need to understand the purpose of patient participation, how to invite patients, how to use plain language, how to manage sensitive information and how to avoid psychological repercussions.

First, there would need to be to be work to raise awareness about the positive aspects of patient participation. (NUR 05)

Showing informational videos could help with adherence. (NUR 06)

Because it's something new, I need to know about it. I need to know who is introducing it. Why and how I should approach it. Training is needed, especially on how to avoid technical terms and what can or cannot be said in front of patients without causing psychological repercussions. (NUR 07)

Another recurrent finding was that professionals believed patients would need explicit encouragement to participate. Participation was described not as spontaneous or automatic, but as something dependent on an explicit opening created by the nursing team.

Maybe during handover we could ask, “Do you have any questions about what we discussed? Would you like to add anything? Did you feel or notice anything about your case?” That could be a way of involving them, either at the beginning or the end of handover. (NUR 05)

#### 4.2.4 Theme 4. Patients' Perceptions of Bedside Handover

This theme was derived from patients' interviews and represented 137 thematic recording units. Patients demonstrated only limited familiarity and superficial knowledge of nursing handover, even though the process occurred at the bedside. They generally understood handover as a conversation among professionals to update the incoming team but did not necessarily recognize it as a moment in which they could participate.

They talk among themselves about the patients' conditions… I think it's important for them to update each other, so care continues, even though everything is written down. (PAT 04)

During handover they explain to each other the patient's problem, like the diagnosis and the medications. (PAT 12)

Patients differed considerably in their willingness to participate. Some did not want to be included because they feared emotional distress, embarrassment or confusion. Others considered that participation might be appropriate only for patients who were clinically and emotionally able to understand and express themselves.

It's not always good. In my case, I'm fine. Thank God, my treatment went well. But for others who didn't have the same success, it could be emotionally distressing. (PAT 01)

It could be interesting for patients who can position themselves properly. But there would need to be some care, because not every patient could participate. It could even confusion them. (PAT 04)

I would feel a little embarrassed, a little ashamed of listening. (PAT 13)

Perhaps the most striking finding was that several patients did not perceive themselves as legitimate participants in handover. They described handover as an activity that belonged to professionals and suggested that the patient's role was limited to receiving care or listening to what professionals decided to share.

I don't think I need to participate. I'm just the patient, it's not my role. What they already tell me is enough. (PAT 05)

No, because I don't have much to say. The meeting is theirs, not mine. What they share with me is already enough. (PAT 11)

In contrast, some patients expressed interest in participating, especially by listening, asking questions, receiving clearer information or complementing information if something was missed.

I'd just like to listen. (PAT 12)

I participated and asked questions. I asked about my case, so I could understand how I am from now on, what I can do and what I cannot do. (PAT 10)

I think it would be interesting to participate. Not that it's necessary, but it could be important, for example, if something is overlooked, the patient could add their perspective. (PAT 04)

Patients also indicated that they would need to be explicitly invited by professionals to feel authorized and comfortable participating.

If they invited me. (PAT 12)

If they came to my bedside, listened to me, and answered my questions. (PAT 15)

Taken together, these findings demonstrate that although bedside handover was routinely performed in the study setting, patient participation remained variable and largely dependent on professionals' communication practices, patients' willingness to engage and the organizational context in which handover occurred.

## 5 Discussion

This study showed that although bedside handover was routinely performed in the coronary intensive care unit, meaningful patient participation remained limited and highly variable. Professionals recognized important benefits of involving patients, whereas patients frequently assumed passive roles unless explicitly invited to contribute. These findings suggest that bedside handover alone is insufficient to ensure participatory communication.

Our findings suggest that bedside handover should not be equated with participatory handover. While bedside handover changes where communication occurs, participatory handover requires changes in how communication occurs by intentionally incorporating patients as active contributors to the exchange of information. This distinction explains why the routine implementation of bedside handover did not consistently result in meaningful patient participation in the present study.

The first theme demonstrated that bedside handover was recognized as an important strategy that facilitated direct patient assessment, verification of clinical information, and reduced the likelihood of omissions during shift changes. These findings are consistent with studies showing that bedside handovers can improve the accuracy of shared clinical information—thereby contributing to safe continuity of care—while also creating opportunities for patient involvement (Hada and Coyer 2021; Tobiano et al. 2018; Chien et al. 2022; Anshasi and Almayasi 2024). However, our findings indicate that these opportunities do not automatically translate into meaningful participation, as patient involvement depends on communication practices that intentionally encourage patients to contribute during handover.

Unlike previous investigations that primarily examined the implementation of bedside handover, this study was conducted in a setting where the practice was already embedded in routine care. This shifts the discussion from whether bedside handover should be adopted to how it can evolve into participatory handover. Although previous studies have highlighted the benefits of bedside handover for information exchange, patient assessment, and individualized care (Kullberg et al. 2018; Chien et al. 2022), our findings demonstrate that bedside handover alone does not ensure patient participation. Patients may be physically present without being invited to ask questions, clarify information, or report concerns. Therefore, physical presence alone should not be interpreted as evidence of meaningful engagement.

Professionals nevertheless recognized that patients could contribute by confirming clinical information, identifying inaccuracies and reporting clinically relevant changes. Similar findings have been reported by Tobiano et al. (2018), Street et al. (2022), and Anshasi and Almayasi (2024), who describe patient participation as an additional safeguard that helps identify omissions or misunderstandings during handover. These findings reinforce that patient participation contributes not only to patient‐centred care but is also a strategy with the potential to improve the quality of communication and reduce the occurrence of missing, incomplete, or incorrect information.

Despite recognizing these benefits, professionals did not consider patient participation appropriate in every clinical situation. Instead, they viewed participation as dependent on patients' clinical condition, communication abilities, emotional readiness, and willingness to become involved. Similar concerns have been described in other settings, where nurses acknowledged the principles of patient‐centred care while questioning the feasibility of involving patients during bedside handover because of workload, organizational demands, and clinical complexity (Khuan and Juni 2017; Paredes‐Garza et al. 2022). Rather than reflecting resistance to innovation, these findings suggest that professionals sought to balance the benefits of participation with their ethical and clinical responsibilities.

This interpretation is consistent with the patient engagement framework proposed by Carman et al. (2013), which conceptualizes engagement as a continuum rather than a dichotomous condition. In the present study, patient participation remained largely at the initial stages of this continuum. Although patients were present and occasionally informed about their care, they were rarely encouraged to actively contribute to communication or share responsibility during handover. Advancing towards participatory handover therefore requires more than relocating communication to the bedside; it requires communication practices that intentionally recognize patients as legitimate contributors to the exchange of clinical information while respecting their preferences and clinical circumstances.

The second theme showed that barriers to patient participation extended beyond operational challenges, encompassing ethical, communicational, and organizational dimensions. Rather than reflecting resistance to change, professionals' concerns revealed the complexity of balancing patient‐centred communication with confidentiality, clinical judgement, and efficient workflow during shift handover. These findings indicate that the principal challenges to participatory handover are relational rather than merely procedural.

Ethical concerns were particularly prominent. Professionals feared that involving patients could expose them to distressing information, increase anxiety, or compromise confidentiality, especially in a coronary intensive care unit where privacy between beds is inherently limited. Similar concerns have been reported in studies of bedside handover, where nurses identified confidentiality, visitors' presence, and discussion of sensitive information as barriers to patient involvement (Kullberg et al. 2018; Clari et al. 2021). However, these concerns should not be interpreted as arguments against participation. Rather, they highlight the need to balance confidentiality with patients' right to receive information and contribute to communication whenever appropriate. Ethical protection and patient participation should therefore be understood as complementary principles.

Communication represented another major barrier. Professionals recognized that technical terminology and clinical jargon could limit patients' understanding, generate unnecessary anxiety, and discourage participation. Similar findings have been reported by Tobiano et al. (2018), Street et al. (2022), and Anshasi and Almayasi (2024), who demonstrated that patient engagement increases when information is communicated in accessible language and patients are explicitly encouraged to ask questions. In our study, concerns about patients' understanding sometimes led professionals to determine what information should or should not be shared before exploring patients' own preferences, potentially reinforcing paternalistic patterns of communication.

These findings should also be interpreted within the Brazilian healthcare context. Although patient‐centred care has progressively gained prominence in national patient safety policies, communication practices in many healthcare institutions continue to be influenced by hierarchical professional relationships and biomedical traditions (Lima and Machado 2021; Yogui et al. 2024). Consistent with Carman et al. (2013), patient engagement depends not only on patients' characteristics but also on communication practices, professional attitudes, and organizational contexts that either facilitate or restrict opportunities for participation.

Organizational constraints further influenced professionals' perceptions of feasibility. Participants described shift handover as a time‐sensitive process and expressed concern that patient participation might prolong communication and disrupt workflow. Similar barriers related to workload, interruptions, and time pressure have been described elsewhere (Khuan and Juni 2017; Clari et al. 2021). Nevertheless, evidence suggests that structured communication strategies enabling patients to confirm information, report concerns, or ask questions can support meaningful participation without compromising efficiency (Chien et al. 2022). Thus, efficiency and patient participation should be regarded as complementary dimensions of high‐quality nursing handover.

Professionals also identified patients' clinical condition as an important determinant of participation. Cognitive impairment, emotional distress, clinical instability, and communication difficulties were perceived as limiting factors, particularly in the intensive care environment (Paredes‐Garza et al. 2022). Rather than justifying exclusion, these findings reinforce that participation should be adapted to each patient's clinical circumstances and preferences.

Collectively, these findings indicate that participatory handover should not be implemented as a standardized requirement. Instead, it should be offered as a flexible communication strategy that combines confidentiality, clinical judgement, and respect for patients' preferences while avoiding both paternalistic exclusion and unrealistic expectations that all patients should participate to the same extent.

The third theme shifted the focus from barriers to the organizational conditions required to implement participatory nursing handover. Rather than questioning whether patients should participate, professionals emphasized the need for organizational structures capable of supporting safe, effective, and patient‐centred communication. This finding suggests that successful implementation depends less on individual willingness than on institutional support, reinforcing participatory handover as a system‐level intervention rather than solely an individual professional responsibility.

Participants consistently highlighted the need for standardized communication processes, protocols, staff training, and leadership support. Similar findings have been reported in studies demonstrating that successful implementation of patient‐centred handover relies on multifaceted organizational strategies rather than isolated behavioural changes (Chien et al. 2022; Clari et al. 2021). Our findings extend this evidence by showing that professionals recognized the need for organizational guidance before participatory handover could become routine practice.

Although professionals supported patient participation, they expressed uncertainty about how to operationalize it in everyday clinical practice, particularly when balancing transparency, confidentiality, emotional protection, and clinical judgement. These findings indicate that implementation should not depend exclusively on individual discretion but should be supported by institutional guidance that promotes consistent practice while preserving professional autonomy in complex clinical situations.

Consistent with Carman et al. (2013), patient engagement depends not only on patients' motivation and capabilities but also on organizational structures that facilitate partnerships between professionals and patients. Accordingly, participatory handover should be understood as an organizational practice embedded within patient‐centred care rather than as an isolated communication technique.

Professionals also recognized that patients require preparation for a more active role during handover. Suggestions such as informational materials, educational videos, explicit invitations to ask questions, and health literacy initiatives may reduce communication asymmetries and strengthen patients' confidence to participate according to their preferences and abilities (Cruz and Pedreira 2020). These findings reinforce that meaningful participation requires intentional facilitation.

Taken together, these findings indicate that participatory handover should be viewed as part of a broader organizational transformation towards patient‐centred care. Consistent with the principles of patient safety culture described by Carvalho and Bates (2025) and Heidmann et al. (2020), this transformation requires leadership commitment, professional education, structured communication processes, and organizational cultures that value dialogue, transparency, and shared responsibility. Embedding participatory communication into routine practice is therefore essential for sustaining patient participation.

The fourth theme explored patients' perceptions of participation in nursing handover and revealed one of the study's most important findings. Although patients valued receiving information about their health condition and appreciated opportunities to communicate with healthcare professionals, many did not perceive themselves as active participants in the handover process. Instead, they largely understood their role as listening, answering questions when requested, and trusting professionals to manage their care. These findings suggest that participatory handover requires changes in both professionals' communication practices and patients' perceptions of their role in care.

This perception is consistent with previous studies demonstrating considerable variation in patients' engagement during bedside handover (Street et al. 2022; Tobiano et al. 2018). While some patients actively ask questions, clarify information, and contribute to communication, many remain passive despite being physically present. Our findings extend this evidence by showing that patients rarely questioned this passive role, suggesting that limited participation may reflect longstanding social expectations regarding the roles of patients and healthcare professionals rather than a lack of interest in participating.

This interpretation is supported by Bressan et al. (2019) and McCloskey et al. (2019), who found that patients frequently associate safe care with confidence in healthcare professionals rather than with their own active involvement. Consistent with Carman et al. (2013), these findings reinforce that patient engagement is a relational process shaped by previous experiences, communication practices, and organizational context. Meaningful participation therefore requires opportunities for involvement and explicit recognition of patients as legitimate contributors to clinical communication.

Trust also emerged as an important component of participation. While confidence in professionals promoted feelings of safety and satisfaction with care, it sometimes reduced patients' perception that their own contributions could improve communication. Trust and participation should be understood as complementary, as trusting relationships may encourage more confident participation (Unbeck et al. 2023).

Patients also differed in their preferred level of involvement. Some wished to receive detailed explanations and actively contribute to discussions, whereas others preferred simply to be informed or to delegate communication to professionals. These findings reinforce that participatory handover should remain flexible, adapting to patients' preferences and clinical circumstances rather than following a standardized model.

Collectively, the findings demonstrate that bedside handover provides the context in which participatory communication may occur but does not, by itself, ensure meaningful patient engagement. This distinction represents the principal conceptual contribution of the present study. Consistent with Carman et al. (2013), engagement should be understood as a continuum in which healthcare organizations progressively create opportunities for patients to move from receiving information to actively contributing to communication.

From an organizational perspective, implementing participatory bedside handover requires complementing the traditional biomedical model with principles of patient‐centred care, shared communication, and respect for patients' preferences. This approach does not diminish professional responsibility or clinical expertise. Instead, it recognizes that technical excellence and patient participation are complementary dimensions of high‐quality and safe nursing care.

This study has some limitations. Data were collected in a single coronary intensive care unit within a public teaching hospital, which may limit the transferability of the findings to other settings. In addition, although both nursing professionals and patients participated, the perspectives of family members and other healthcare professionals were not explored. As a qualitative study conducted in a single organizational context, the findings should be interpreted as context‐specific rather than universally transferable. Future research should examine participatory handover in different healthcare settings, investigate implementation strategies, and evaluate how organizational interventions influence patient engagement, communication processes, and patient safety over time.

### 5.1 Implications for Nursing Practice

This study offers both conceptual and practical contributions to nursing practice. By exploring the perspectives of nursing professionals and patients in a setting where bedside handover was already established, it advances understanding of the transition from bedside communication to participatory communication. The study demonstrates that bedside handover and participatory handover should not be regarded as synonymous practices. These findings provide practical guidance for implementing patient‐centred handover, aiming to strengthen partnerships between professionals and patients and promote patient safety.

## 6 Conclusion

This study demonstrated that implementing patient participation in nursing handover involves more than performing handover at the bedside. Although nursing professionals recognized the potential benefits of patient participation for communication, patient safety, and continuity of care, they also identified ethical, communicational, and organizational challenges that influence its implementation. At the same time, patients valued receiving information but often did not perceive themselves as legitimate participants in the handover process, reflecting the persistence of traditional professional‐centred roles.

The main contribution of these findings is to demonstrate that bedside handover and participatory handover should not be regarded as synonymous practices. Rather, bedside handover creates the context in which participatory communication may occur, whereas meaningful participation depends on communication practices that intentionally recognize patients as legitimate contributors to the exchange of clinical information.

The findings indicate that the transition from bedside handover to participatory handover requires supportive organizational environments, effective communication strategies, and a culture that fosters partnerships between professionals and patients. Rather than adopting a standardized model, participation should be tailored to patients' preferences, communication abilities, health literacy, and clinical condition, ensuring that every patient has the opportunity, but not the obligation, to participate.

## Author Contributions

**Grazielle Rezende da Silva dos Santos:** conceptualization, investigation, writing – original draft, methodology, formal analysis, project administration, data curation. **Sabrina da Costa Machado Duarte:** funding acquisition, writing – review and editing, formal analysis, methodology, writing – original draft. **Juliana Faria Campos:** writing – review and editing, validation, methodology, formal analysis, writing – original draft. **Rafael Celestino da Silva:** conceptualization, funding acquisition, writing – original draft, writing – review and editing, methodology, formal analysis, project administration, supervision.

## Funding

This study was carried out with the support of the Research Foundation of Rio de Janeiro (FAPERJ), under protocol no. E‐26/010.001278/2019, and Coordination for the Improvement of Higher Education Personnel—Brazil (CAPES)—Funding Code 001.

## Conflicts of Interest

The authors declare no conflicts of interest.

## Supporting information


**Data S1:** Interview script—nursing professionals.

## Data Availability

The data that support the findings of this study are available from the corresponding author upon reasonable request.
