# Supplementary material for: Patient Participation in Nursing Handover in a Coronary Intensive Care Unit: A Qualitative Study
Source: Nurs Open. 2026 Sep 8;13(9):e70802. doi: 10.1002/nop2.70802 (PMC13553063; doi:10.1002/nop2.70802)
Supplement: Supplementary file 1 [file NOP2-13-e70802-s001.docx]

# **INTERVIEW SCRIPT – NURSING PROFESSIONALS**

1-Could you tell me about how the nursing handover during shift changes is performed in this sector in relation to patient safety?

2-How would you rate patient participation in the shift change nursing handover in this sector?

3-What are your thoughts on a patient-participated nursing handover model in terms of safety? What would be its advantages and disadvantages?

4-Do you believe there are difficulties in promoting patient participation during shift change nursing handovers? If so, what are they? Is there anything that can be done to resolve them? If so, what?

5-In your opinion, how important is patient involvement and participation in their care for promoting safety?

6-Do you think professionals can do anything to promote patient participation during nursing handover in this sector?

7-Are you in favor of patient/family participation in nursing handover during shift changes? If yes, why? If no, why?

8-And what do you think your colleagues' position is on patient participation in shift change nursing handovers?

9-In your assessment, what would be the role of the patient/family member in communicating the clinical information necessary for the continuity of nursing care?

10-How do you think the nursing handover model with patient participation should be? What information should be included? Is there a specific moment when you would like the patient to participate during information sharing? At what moments would you not want their participation?

11-In your opinion, what would be necessary for the implementation of a new nursing handover model with patient participation?

12-Do you feel prepared to promote patient participation during nursing handover? If so, why? If not, what are you lacking?

13-Are there any actions that could be taken in the sector to encourage patient /family participation in nursing handover?

**INTERVIEW SCRIPT – PATIENTS**

1-What do you know about the nursing handover that occurs during shift changes in this sector?

2- How would you rate the patient's participation during communication between nursing professionals in the handover?

3- In your opinion, should the patient participate in the nursing handover? If yes, why? If no, why?

4- Would you like to participate in the handover by providing nursing professionals with information about your health? If so, how would you participate?

5- If the professionals were to perform the nursing handover at your bedside and give you the opportunity to participate, what would you like to ask?

6-What would patients need to know to participate in the shift change nursing handover?

7-What would encourage/facilitate your participation? And what would not encourage/facilitate it?

8-Is there any type of information you would like to hear from nursing professionals? What information would you not want shared in front of you?

9-Do you feel ready to participate in the shift change nursing handover? If yes, why? If not, why?
